# The Eukaryotic Elongation Factor 1A Is Critical for Genome Replication of the Paramyxovirus Respiratory Syncytial Virus

**DOI:** 10.1371/journal.pone.0114447

**Published:** 2014-12-05

**Authors:** Ting Wei, Dongsheng Li, Daneth Marcial, Moshin Khan, Min-Hsuan Lin, Natale Snape, Reena Ghildyal, David Harrich, Kirsten Spann

**Affiliations:** 1 Queensland Institute of Medical Research Berghofer, Herston, Australia; 2 Clinical Medical Virology Centre, The University of Queensland, Herston, Australia; 3 Sir Albert Sakzewski Virus Research Centre, Childrens Health Queensland, Herston, Australia; 4 Centre for Research in Therapeutic Solutions, University of Canberra, Canberra, Australia; 5 Australian Infectious Disease Research Centre, St Lucia, Australia; University of Georgia, United States of America

## Abstract

The eukaryotic translation factor eEF1A assists replication of many RNA viruses by various mechanisms. Here we show that down-regulation of eEF1A restricts the expression of viral genomic RNA and the release of infectious virus, demonstrating a biological requirement for eEF1A in the respiratory syncytial virus (RSV) life cycle. The key proteins in the replicase/transcriptase complex of RSV; the nucleocapsid (N) protein, phosphoprotein (P) and matrix (M) protein, all associate with eEF1A in RSV infected cells, although N is the strongest binding partner. Using individually expressed proteins, N, but not P or M bound to eEF1A. This study demonstrates a novel interaction between eEF1A and the RSV replication complex, through binding to N protein, to facilitate genomic RNA synthesis and virus production.

## Introduction

Human translation elongation factor eEF1A, is a subunit of the eukaryotic translation elongation 1 complex (eEF1) [1]. This complex delivers aminoacylated tRNA to the elongating ribosomes during protein synthesis. In addition, eEF1A binds to RNA and actin in non-canonical cellular roles including protein degradation, apoptosis, nucleocytoplasmic trafficking, heat shock responses and cytoskeletal regulation. Several RNA viruses also utilize eEF1A for replication, although the mechanisms by which they do this are varied [2]–[7].

The virions of non-segmented negative-strand RNA viruses, (Mononegavirales order) contain a virally-encoded RNA-dependent-RNA polymerase (RdRp; also called L protein), which transcribes viral genomic RNA into discrete mRNAs, which encode viral proteins. The RdRp also synthesizes full-length positive-strand antigenome which is used as a template for the synthesis of new negative-strand genomes. A rhabdovirus from this order, vesicular stomatitis virus (VSV), forms two RdRp complexes; the transcriptase which incorporates eEF1A and a virus-encoded phosphoprotein (P), and the replicase which does not incorporate eEF1A, but rather the P and a virus-encoded nucleocapsid (N) protein [8], [9]. Therefore eEF1A is required to synthesize capped VSV mRNA, but is not required to synthesize viral genomic RNA.

Respiratory syncytial virus (RSV) is a member of the *Paramyxoviridae* family, also from the mononegavirales order, and causes respiratory disease in infants, young children and the elderly worldwide [10]–[12]. Despite decades of research there are currently no targeted antiviral therapies and treatment is limited to relief of symptoms and expensive monoclonal antibody treatment [13], [14]. One possible strategy to inhibit RSV infections is to identify and target viral dependency factors, such as cellular proteins that make virus replication in cells possible. RSV ribonucleoprotein (RNP) complexes are composed of viral RNA, the proteins L (the RSV RdRp), N and P, and are responsible for viral replication and transcription [15]. Therefore we were interested to know if, like VSV, RSV incorporated eEF1A in the RNP complex, and if this was required for viral replication. To our knowledge, a specific role for eEF1A in the replication of any paramyxovirus is unknown.

## Materials and Methods

### Downregulation of eEF1A using siRNA

siRNA molecules targeting the eEF1A mRNA transcript (ID: SASI_Hs02_00331772 and SASI_Hs02_00331773, Sigma-Aldrich) or a universal siRNA negative control (siMM, ID: SIC001, Sigma-Aldrich) were transfected into HEK293T cells with Lipofectamine RNAiMAX according to the manufacturer's protocol (Life Technologies). An additional control of untreated cells was included. Cells were incubated with siRNA molecules at 37°C for 48 h to ensure maximal down-regulation of eEF1A prior to RSV infection. Three replicate cultures of untreated, siMM or sieEF1A siRNA-transfected cells were maintained in parallel with RSV-infected cultures for the infection period of a further 48 h. The efficiency of eEF1A down-regulation was quantified by western blot analysis and densitometry using ImageJ [16]. Cell viability following transfection was determined using CellTitre 96 Aqueous One solution (Promega).

### Virus infection

RSV (A2; ATCC) was propagated in human epithelial carcinoma cells (HEp-2a) and a stock for infection experiments was purified through a sucrose cushion [17]. The viral titer of the stock was quantified by standard immune-plaque assay [17]. Briefly, a 10-fold dilution series of virus suspension was used to infect Hep-2a monolayers and overlaid with 0.8% methyl cellulose. Following 6 days incubation at 37°C, monolayers were fixed with 60% methanol/40% acetone, blocked with 5% skim milk/PBS and probed with a goat-anti RSV polyclonal antibody (Virostat). HRP-conjugated secondary antibody and DAB colour developer (Sigma-Aldrich) was then used to visualise RSV-positive plaques and calculate viral titre as plaque forming units (pfu)/ml. HEK293T cells or human lung epithelial cell carcinoma (A549) cells were infected with RSV at either a multiplicity of infection (MOI) of 1 or 0.1 pfu/cell.

### RNA extraction and qPCR

Total RNA was extracted from cells using TRIzol (Life Technologies), and 500 ng total RNA was used to generate cDNA with either oligo-dT_(21)_ or random hexamer (Life Technologies) primers. qPCR was performed using dual labeled probe/primer sets (Sigma-Aldrich) specific for N mRNA or the N-P region of the RSV genome, and also cellular β-actin as an internal control (Table 1). mRNA and genomic RNA were expressed as a fold induction over uninfected cells using 2^−ΔΔCt^.

**Table 1:** Primer and probe sequences used for quantitative PCR.

TARGET	Primer/probe	Sequence 5′ – 3′
RSV N mRNA	Forward primer	TGGGAGAGGTAGCTCCAGAA
	Reverse primer	AGATCTGTCCCCTGCTGCTA
	Probe	HEX-GCATGACTCTCCTGATTGTGGGATGATA-BHQ1
RSV genomic RNA	Forward primer	TGACAGCAGAAGAACTAGAGGCT
	Reverse primer	TTTGGGTGATGTGAATTTGC
	Probe	HEX-TTCCATGGAGAAGATGCAAACAACAGG-BHQ1
β-actin	Forward primer	GGCATCCACGAAACTACCTT
	Reverse primer	AGCACTGTGTTGGCGTACAG
	Probe	FAM-ATCATGAAGTGTGACGTGGACATCCG-BHQ1

### Western Blot Analysis

Cell lysates were prepared in Pierce lysis buffer (Thermo Scientific) and total protein was quantified using a Bradford assay (Bio-Rad). Proteins were separated by SDS-PAGE, transferred to PVDF membranes and probed using rabbit anti-eEF1A polyclonal (Santa Cruz), goat anti-RSV polyclonal (Virostat) to detect RSV N, P and M, mouse anti-FLAG monoclonal (Sigma-Aldrich) to detect FLAG-tagged M protein, or mouse anti-β-tubulin as a loading control.

### Proximity Ligation Assay

Uninfected (control) and RSV-infected (MOI 1 pfu/cell) A549 cells were fixed with cold acetone/methanol (1∶1) 24 h after infection and proximity assays performed using the Duolink II system and PLA probes (Olink Bioscience). Monoclonal primary antibodies specific for eEF1A (Santa Cruz) and either RSV N or P proteins (Abcam) were allowed to bind *in situ* to proteins within fixed and permeabilized cells on a chamber slide. Then, secondary antibodies conjugated with oligonucleotides (PLA probe PLUS and PLA probe MINUS) were added. The two PLA probes ligate if they are in close proximity, and allow a polymerase-driven amplification reaction of fluorescent-labeled oligonucleotides. Images were taken using a confocal microscope and the number of positive foci quantified for 50 cells/reaction using the Duolink Imagetool software (Olink Biosciences). Antibodies to the human transcription initiation factor eIF3A were used as a negative control for proximity to RSV N.

### Transfection of RSV N, P and M expression plasmids

pTM plasmids expressing either RSV N or P (a gift from Peter Collins) were used to transfect BSR-T7 cells [18] using Lipofectamine 2000 (Life Technologies). pCAG-M-FLAG, expressing RSV M, was used to transfect HEK293T cells. An empty FLAG vector was also used to transfect HEK293T cells as a negative control. Cells were lysed 48 h post-transfection for co-immunoprecipitation (co-IP).

### Co-Immunoprecipitation

RSV-infected HEK293T and A549 cells, or cells transfected and expressing RSV N, P or M proteins were lysed in S100 buffer containing 0.3% Tween-20 and then incubated with Dynabeads M-270 Epoxy beads (Life Technologies) coupled to anti-eEF1A antibody (Santa Cruz) for 1 h at 4°C with rotation. Cell lysates were also incubated with uncoupled beads, or beads coupled to BSA or antibody to a control eIF3A (Santa Cruz). Beads were washed 3 times in PBS and the proteins were eluted by boiling in 1× SDS-PAGE loading buffer. Eluted proteins were separated by SDS-PAGE electrophoresis and western blot analysis was performed to detect immunoprecipitated eEF1A and RSV N, P and M proteins.

### Statistical analysis

Data were analysed for statistical differences using either paired Welch's t test from at least 3 independent experiments or measurements, or two way ANOVA.

## Results

### eEF1A is required for infectious virus release

**Figure 1:**
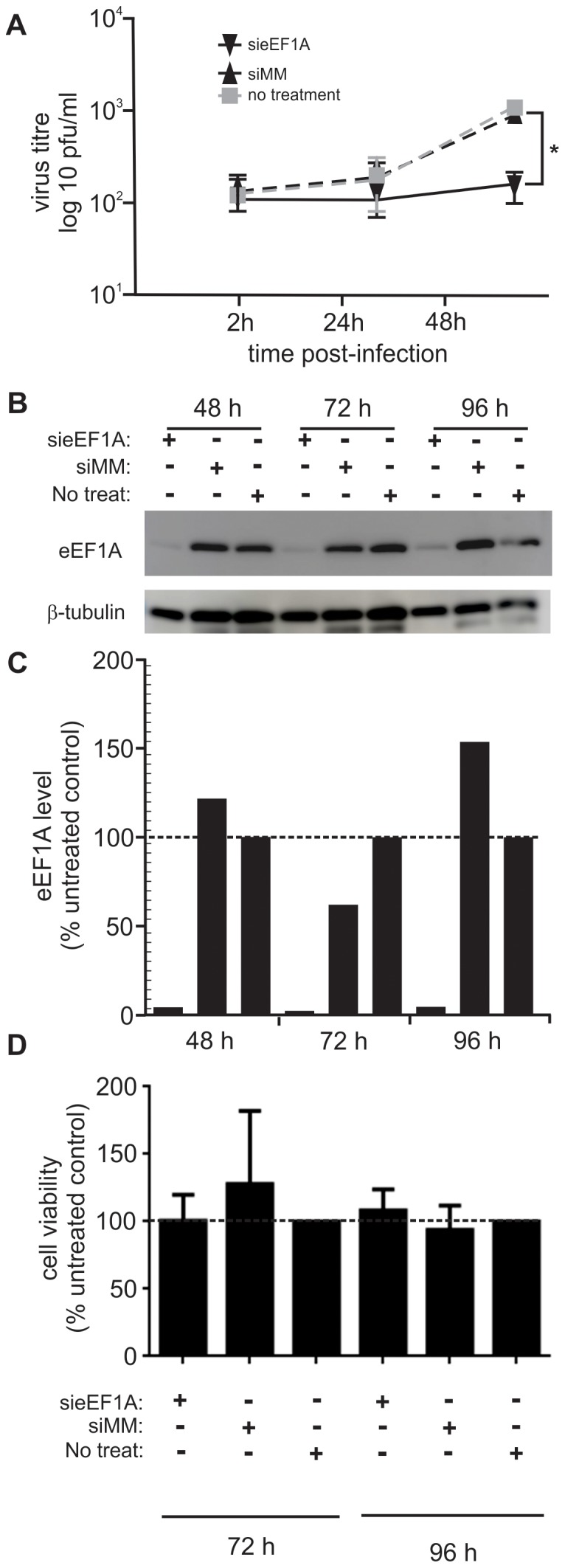
Down-regulation of eEF1A reduces the amount of infectious virus released from RSV-infected cells. HEK293T cells were transfected with sieEF1A, or a non-specific control siRNA (siMM), 48 h prior to infection with RSV A2 at a MOI of 0.1 pfu/cell. Infectious virus released from the cells into the culture supernatant was quantified by immune-plaque assay using Hep-2a cells exposed to culture supernatants, then overlayed with methyl cellulose and incubated at 37°C for 6 days. RSV-positive plaques were detected using antisera to RSV. (**A**) The amount of infectious virus released by cells in which eEF1A had been down-regulated was significantly reduced 48 h post-infection, compared to cells that had been transfected with the siMM control or untreated. (**B**) Down-regulation of eEF1A>90% by sieEF1A and not the siMM control was confirmed by western blot analysis of cell lysates at the time of infection, which was 48 h after transfection and also for the 24 h and 48 h following RSV infection (72 h and 96 h post-transfection). Beta-Tubulin was used as a loading control. (**C**) A digitized western blot in (B) was analyzed using ImageJ software. The eEF1A level (average pixel intensity) in each lane was normalized to the corresponding level of β-tubulin in the same lane. For each time point, the amount of eEF1A detected in untreated control samples was designated as 100%. (**D**) HEK293T cells transfected with either sieEF1A or siMM, remained viable compared to untreated cells 72 h and 96 h post-transfection. The average cell viability compared with an uninfected control was calculated. siRNA experiments were repeated three times with similar results. Mean values, with SEM are shown. Significance identified using paired t-test. *P<0.05

We previously used siRNA to down-regulate eEF1A in HIV-1 infected cells to demonstrate that eEF1A is a component of the HIV-1 reverse transcriptase complex and an important factor in HIV-1 early replication [19]. We used the same siRNA (called sieEF1A), or a mismatch siRNA (siMM) molecule as a negative control, to down-regulate eEF1A in HEK293T and A549 cells. Down-regulation of eEF1A was limited in A549 cells, hence HEK293T cells were used in this study. Transfected and untransfected HEK293T cells were infected with RSV at a MOI of 0.1 pfu/cell 48 h following transfection. The amount of infectious virus shed into cell culture supernatant over the subsequent 48 h was quantified by performing immune-plaque assay for three independent experiments. Down-regulation of eEF1A resulted in a significant (*P*<0.05) reduction of viral titres 48 h post-infection (p.i.) compared to cells either not transfected or transfected with siMM (Figure 1A). While siMM or untransfected cells shed 10-fold more RSV at 48 h compared to 2 h post-infection, sieEF1A-transfected cells shed very little virus. Western blot analysis demonstrated that eEF1A down-regulation was sustained for the 48 h p.i. (96 h post-transfection; Figure 1B and 1C). Cell viability was also not affected by transfection with either sieEF1A or siMM, compared to non-transfected cells at 72 and 96 h post transfection (Figure 1D). Although the amount of virus shed from infected and untreated HEK293T cells is less that would be expected from A549 cells [17] these data do demonstrates a biological dependency of RSV on eEF1A for the efficient shedding of infectious virus.

### eEF1A is required for efficient viral genome replication

**Figure 2:**
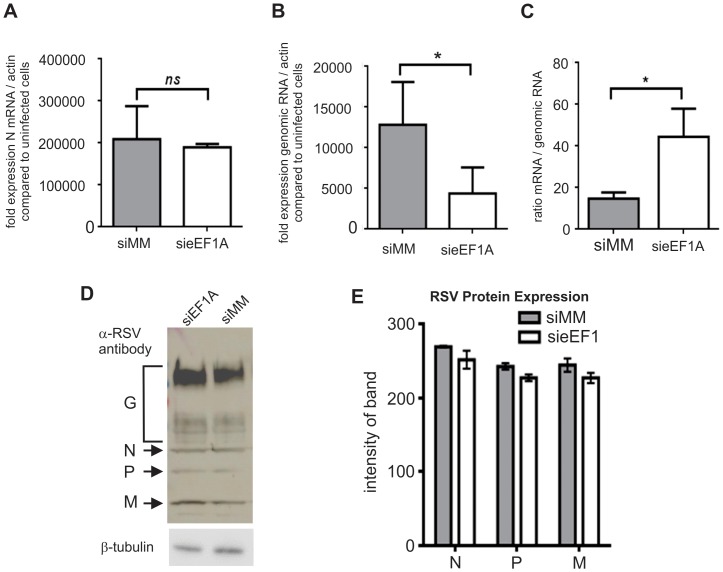
Down-regulation of eEF1A reduces RSV genome expression, but not mRNA transcription or protein expression. HEK273T cells were infected with RSV at a MOI of 0.1 pfu/cell. (**A**) The transcription of RSV nucleocapsid (N) mRNA, was quantified by qRT-PCR using dual-labelled probes specific for RSV N and β-actin. N mRNA was expressed as fold induction compared to uninfected cells, and normalized to β-actin expression. Down-regulation of eEF1A did not affect RSV N mRNA transcription. (**B**) Down-regulation of eEF1A significantly reduced replication of RSV genomic RNA, as quantified by qRT-PCR using dual-labeled probes specific for the N-P (phosphoprotein) region of the RSV genome and β-actin. RSV genomic RNA was expressed as fold induction compared to uninfected cells, normalized to β-actin expression. (**C**) The ratio of viral mRNA/genomic RNA was calculated using the same data as in A and B, and identified a trend towards elevated viral mRNA relative to genomic RNA when eEF1A is down-regulated. (**D**) RSV protein expression was not affected by down-regulation of eEF1A, as demonstrated by Western blot analysis of SDS-PAGE separated proteins using an anti-RSV polyclonal antibody that detected RSV N, P and M. β-tubulin was used as a loading control. (**E**) Densitometry was performed using the Odyssey (Li-Cor) to quantify signal intensity of the RSV N, P and M proteins detected by western blot analysis and normalised against β-tubulin expression. Experiments were repeated three times. Mean values, with SEM are shown. Significance identified using paired t-test. *P<0.05

To determine if the down-regulation of eEF1A affected RSV mRNA transcription, genomic RNA replication or protein expression, we quantified RSV N mRNA transcripts and RSV genomic RNA expressed in the cells at 48 h p.i. Down-regulation of eEF1A did not affect viral N mRNA transcription (Figure 2A), however it did significantly reduce (*P*<0.05) detectable viral genomic RNA (Figure 2B). This measure would be representative of intracellular progeny genome and also viral particles associated with the plasma membrane prior to complete assembly and egress. When the same data were re-analysed as a ratio, a significant increase (*P*<0.05) in the ratio of N mRNA/genomic RNA was detected when eEF1A expression was reduced (Figure 2C). SDS-PAGE separation of viral proteins from cell lysates and western blot analysis using anti-RSV polysera showed that down-regulation of eEF1A did not affect levels of RSV protein 48 h p.i. (Figure 2D and E). These data indicate that down-regulation of eEF1A by siRNA did not affect viral gene transcription or translation into protein. However it did affect genome replication and the subsequent release of infectious virus. It is feasible to assume that beyond 48 h p.i., the reduction in shed infectious virus and genomic RNA would also cause a reduction in viral mRNA transcription and protein production. Down-regulation of eEF1A beyond this time was not sustained (not shown), therefore we could not investigate the effect of eEF1A down-regulation beyond 96 h post-transfection.

### eEF1A is associated with viral replication complex proteins in RSV infected cells

**Figure 3:**
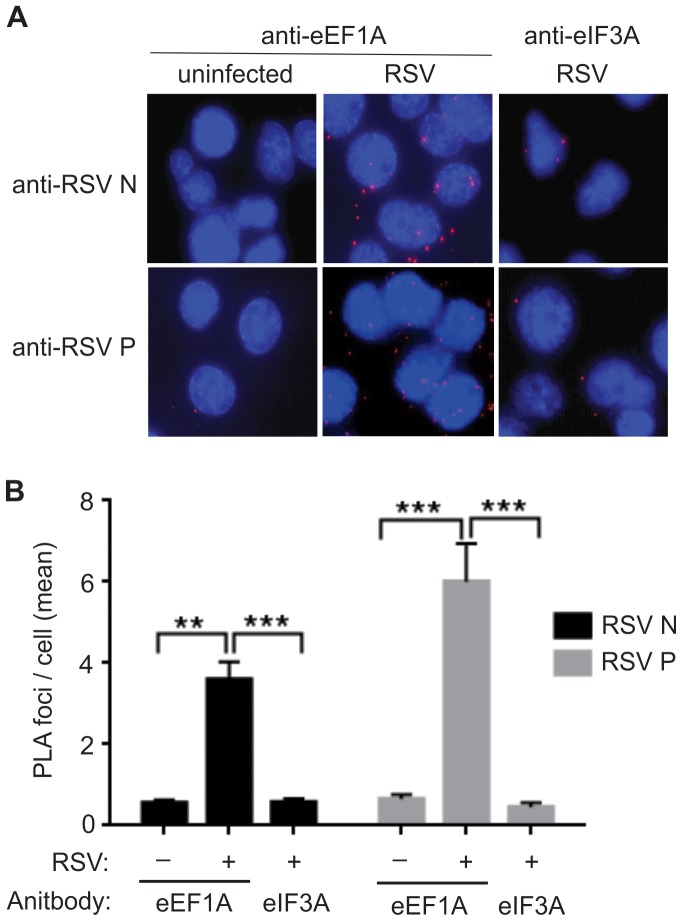
Nucleocapsid (N) and phosphoprotein (P) co-localise with host eEF1A in a live virus infection. A549 cells were infected with RSV at a MOI of 1 pfu/cell and fixed for proximity assay 24 h post-infection. Antibodies to eEF1A or eIF3A (negative control), and RSV N and P were used in conjunction with Duolink II PLA probes to detect significant proximity between RSV N, P and eEF1A, and no significant proximity between RSV N, P and eIF3A. (**A**) Images were captured on a confocal microscope and (**B**) the number of signals (foci) detected in 100 cells per reaction was quantified using the Duolink Imagetool software (Olink Biosciences). Data was collected by two readers and the mean number of signals/cell was then calculated. Experiments were repeated three times. Mean values, with SEM. Significance identified using 2-way ANOVA. **P<0.05, ***P<0.001.

A proximity ligation assay (PLA) was performed to demonstrate the association of eEF1A with the N and P proteins of the RSV replication complex. RSV-infected A549 cells were fixed at 24 h p.i. and the PLA assay was performed using anti-eEF1A and anti-N, or anti-eEF1A and anti-P antibodies. As a control, anti-eEF1A antibody was replaced with anti-eIF3 antibody. A significant number of ligation foci for RSV N or P with eEF1A (*P*<0.001) were identified in infected cells, compared to ligation foci for RSV N or P with eIF3 (Figure 3A and B). The numbers of ligation foci for RSV N or P with eIF3 were similar to the un-infected cells probed with anti-eEF1A and N or P, suggesting a level of background antibody interaction (Figure 3A and B). This result suggested a strong interaction between eEF1A and the N and P proteins, most likely within RSV RNP complex in the infected cells.

**Figure 4:**
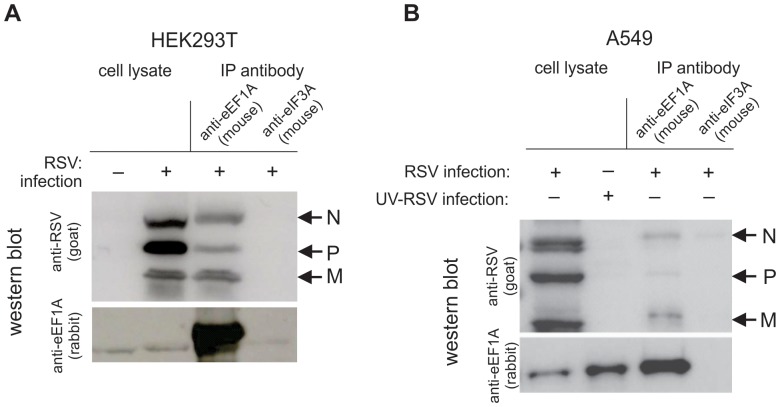
RSV nucleocapsid (N), phosphoprotein (P) and matrix (M) bind to eEF1A in a live virus infection. (**A**) HEK293T or (**B**) A549 cells were infected with RSV at a MOI of 1 pfu/cell and lysed 48 h post-infection. The lysate was incubated with beads bound with antibodies to either eEF1A or eIF3A (negative control). Western blot analysis was performed on lysates before and after immunoprecipitation using antibodies to RSV or eEF1A. A representative blot of immunoprecipitation performed 3 times with consistent results is shown.

In order to investigate if eEF1A is directly associated with the N and P proteins of the replication complex, co-IP assays were performed using cell lysates of both HEK293T and A549 cells infected with RSV at a MOI of 1 pfu/cell. Although reliable down-regulation of eEF1A was not achieved in A549 cells, we investigated the interactions of eEF1A and RNP complex proteins in this cell line, as a more biologically relevant cell line for RSV studies. Bound protein complexes were eluted and separated by SDS-PAGE. Western blot analysis showed that RSV N and P were co-immunoprecipitated with eEF1A in both HEK293T cells (Figure 4A) and A549 cells (Figure 4B), indicating that eEF1A effectively interacts with the RSV RNP complex in both cell lines. Interestingly, RSV M protein also bound to eEF1A in both cell types. It is likely that eEF1A interacts with N, P and M in complex during viral replication/transcription.

### eEF1A binds to the RSV N, but not P and M proteins when expressed individually

**Figure 5:**
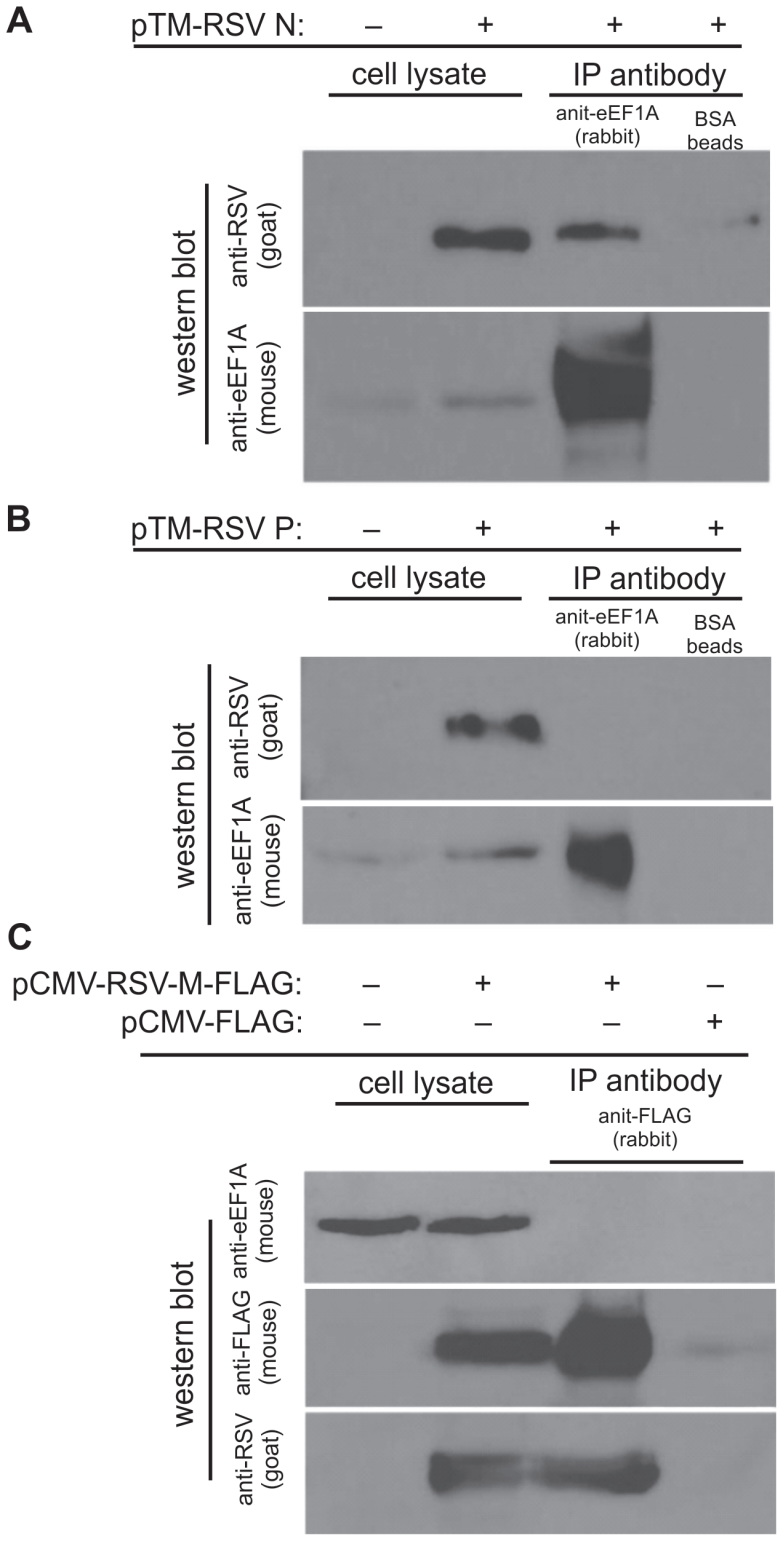
Nucleocapsid (N), but not phosphoprotein (P) or matrix (M) protein bind to eEF1A when expressed singly from plasmids. BSR-T7 cells were transfected with plasmids expressing either (A) RSV N or (B) RSV P and immunoprecipitation performed using beads either unbound (beads only) or bound with antibodies to eEF1A. Lysates before and after immunoprecipitation were separated by SDS-PAGE and probed using a polyclonal anti-RSV antibody and an anti-eEF1A antibody. (C) HEK293T cells were transfected with a plasmid expressing FLAG-M and immunoprecipitation performed using beads either unbound (beads only) or bound with antibodies to FLAG. Lysates before and after immunoprecipitation were separated by SDS-PAGE and probed using an anti-RSV polyclonal antibody, anti-FLAG antibody, or anti-eEF1A antibody.

We then investigated which RSV proteins bind directly to eEF1A in the absence of replicating virus. The N, P and M proteins were individually expressed in cells, and co-IP performed using anti-eEF1A or anti-flag antibody-coupled Dynal beads from the cell lysates. Here we used BSR-T7 cells to maximise the expression of RSV N and P proteins. The M protein was tagged with flag for the purpose of co-IP. The results show that eEF1A bound directly to RSV N protein (Figure 5A), but not to the P (Figure 5B) or M (Figure 5C) proteins. This suggests that within the RSV RNP complex, eEF1A interacts directly with the N protein, and that interaction with the P and M proteins may be weak or indirect.

## Discussion

The eukaryotic translation elongation factor 1A (eEF1A) is part of the translation elongation factor 1 complex that also contains valyl-tRNA and eEF1B, which itself is comprised of eEF1G, eEF1B and eEF1D. The role of eEF1B is to exchange GDP for GTP on eEF1A so that it can bind and deliver aminoacylated transfer RNA (aa-tRNA) to the elongating ribosome. eEF1A is an abundant and multifunction protein as it also plays a non-classical role in nucleocytopalsmic trafficking [20], [21], protein degradation [22], heat shock [7], [23] and apoptosis [23]. RNA viruses have subverted eEF1A for viral replication via different mechanisms. eEF1A has been found in association with viral genomic RNA [24]–[26], and also with viral transcription/replication complex proteins [27]–[29]. The interactions of eEF1A with viral RdRp have been studied in several positive sense RNA viruses, including the plant RNA viruses tobacco mosaic virus (TMV) [28], [30] and tomato bushy stunt virus (TBSV) [29], [31]. However studies for negative sense RNA viruses are fewer. This is the first report that eEF1A is important for genome replication and the release of infectious virus for the negative sense RNA virus RSV. VSV, and now RSV are the only reported negative sense viruses that utilize eEF1A in transcription or replication. However, VSV and RSV differ in the mechanisms by which they utilise eEF1A. Our data suggest that eEF1A may be more involved in replication of RSV than transcription. However for VSV, transcription is initiated by eEF1A is complex with the polymerase, while replication is initiated by a L-N-P complex without eEF1A [9]. Again, this indicates the diverse mechanisms by which viruses subvert eEF1A.

Here we focused on the role of eEF1A, rather than eEF1G, in RSV replication, as the overwhelming majority of published research suggests that eEF1A is the principal functional cellular partner involved in viral replication. We have previously shown an association between HIV-1 reverse transcriptase and eEF1G [19]. However, more recent data from our lab has shown that eEF1A is the principal effector in early HIV-1 replication, and a function for eEF1G will require further investigation (Li et al., submitted). eEF1Bγ is the plant homolog of eEF1G, and is important for RNA synthesis by the TBSV replicase [32]. However there are no other reports of eEF1G involvement in animal RNA virus replication.

Our co-IP studies demonstrated that RSV N binds directly to eEF1A, and indirectly to P within the RNP. We cannot rule out the possibility that eEF1A binds directly to the viral polymerase (L), although the molecular tools to investigate this aspect are not readily available. We have also shown an indirect association between eEF1A and M. As the M protein is involved in virion assembly, this is not surprising. Indeed, the M protein has previously been co-precipitated with N [33]. RSV M is also thought to interact with the RNP to inhibit the transcriptase activity of the nucleocapsid prior to assemble [34], [35].

Similarly to our observations here with RSV, down-regulation of the eEF1A plant homolog by gene silencing significantly reduced viral RNA levels and the spread of TBSV infection [30]. This has also been demonstrated using eEF1A chemical inhibitors didemnin B and gamendazole, which significantly reduced viral replicase activity by TBSV [29], [31]. As eEF1A is critical for many cellular processes, targeting eEF1A as an antiviral strategy would be challenging. However disrupting the viral subversion of eEF1A without affecting the essential functions of eEF1A in the cell may be a promising area for further research.
